# “Do I want to know it all?” A qualitative study of glioma patients’ perspectives on receiving information about their diagnosis and prognosis

**DOI:** 10.1007/s00520-020-05846-7

**Published:** 2020-10-30

**Authors:** Annika Malmström, Lisa Åkesson, Peter Milos, Munila Mudaisi, Helena Bruhn, Michael Strandeus, Marit Karlsson

**Affiliations:** 1 Department of Advanced Home Care, Linköping University, 58185 Linköping, Sweden; 2 Department of Biomedical and Clinical Sciences, Linköping University, 58185 Linköping, Sweden; 3 Regional Cancer Center South East, Region Östergötland, 58185 Linköping, Sweden; 4 Department of Neurosurgery, Linköping University, 58185 Linköping, Sweden; 5 Department of Oncology, Linköping University, 58185 Linköping, Sweden; 6 Department of Neurology, Ryhov Hospital, 55305 Jönköping, Sweden; 7 Department of Oncology, Ryhov Hospital, 55305 Jönköping, Sweden

**Keywords:** Qualitative study, Glioma, Information on diagnosis, Information on prognosis, Patient-accessed electronic health record

## Abstract

**Purpose:**

Glioma patients have poor prognosis. The amount of detail of disease-related information patients wish to receive is not known. The aim of this study was to explore glioma patients’ experiences and preferences regarding receiving information on diagnosis and prognosis.

**Methods:**

Semi-structured interviews were performed with patients diagnosed with glioma. The interviews were analysed by qualitative content analysis without predefined categories by two independent coders.

**Results:**

Ten women and 15 men, with newly diagnosed grade II–IV glioma, age 25–76 years, were interviewed. Participants’ experience on diagnosis communication was either *indirect*, meaning they found out their diagnosis unintentionally, e.g., from their electronic health record (EHR) instead of from their doctor, this causing anxiety and feelings of abandonment, *insufficiently tailored*: lacking in many aspects or *individualised and compassionate.* Participants generally wanted to know “the truth” about diagnosis and prognosis, but what they meant varied; some desired full honest information to allow for autonomous choices, others preferred general information without details, and some wanted no bad news at all, only positive information. Participants disclosed vulnerability after receiving their diagnosis, being cast into the unknown. They expressed a need for better everyday practical information to help create some control. Supportive staff could reduce participants’ distress.

**Conclusion:**

There is a need to further develop and implement individually tailored information to glioma patients, both in consultations and patient-accessed EHR systems, which should have safe guards for sensitive information. Not all patients want to know it all, one size does not fit all.

**Supplementary Information:**

The online version contains supplementary material available at 10.1007/s00520-020-05846-7.

## Introduction

Malignant gliomas will decrease life expectancy, although glioblastomas (GBM) and low-grade gliomas (LGG) differ regarding prognosis and treatment and affect different age groups [1–6]. They cause neurological and cognitive decline and threaten the patient’s personality along the disease trajectory. At the time of primary investigation, the health practitioner needs to provide information to patients about their diagnosis, prognosis and treatment options. This is expected to be perceived as bad news by the patient.

In Sweden, patients are according to law to be informed honestly and fully about their disease and treatment options [7]. In contrast to this, several studies in cancer show that, to preserve hope, not all patients want full information when the prognosis is pessimistic [8–10].

How health practitioners should provide information to glioma patients requires further investigations. A better understanding of the patient’s perspective is expected to improve their care.

This study was designed to explore how glioma patients wish to be informed about diagnosis and prognosis, also if, for example, no therapy that could influence the disease was available. We also investigated their experiences and reflections regarding recently received information on diagnosis and prognosis.

## Methods

Data was collected through interviews with newly diagnosed glioma patients, recruited in the postoperative period after primary surgery at the Neurosurgery, Oncology or Neurology Department before start of oncological treatment. Participants were selected through maximum variation sampling according to gender, age and tumor type (both GBM and LGG), in order to explore rich data on preferences and experiences from a diverse patient group [11]. Exclusion criteria were mentally or physically unable to participate, as judged by their physician, or being non-Swedish speaking. Patients accepting participation were contacted by phone by the interviewer, to set a time and place for the interview. The place, chosen by the participant, was most often their home, otherwise the hospital. Most were interviewed before start of postoperative treatment. In all, 39 patients agreed to be contacted. Fourteen were not interviewed due to fast deterioration [5], changing their mind about participation [1], logistic or sampling reasons [8]. All signed written informed consent before participation. The study was approved by the Regional Ethics Committee.

Before the interview started, we screened for factors that could influence the interview, such as symptom burden or cognitive decline. Therefore, the participants filled out the European Organisation for Research and Treatment of Cancer (EORTC) QLQ-C30 quality of life (QoL) questionnaire with Brain Cancer Module BN20 [12–14], assessing different aspects that define QoL in brain tumor patients. For cognitive assessment, the MOCA test, a quick and simple tool testing various cognitive functions, was carried out [15–17].

### Interviews

All 25 interviews were audio-recorded and conducted between April 2016 and October 2017 by two authors, MK or AM. They varied in length from 16 to 75 min. The participants were interviewed unaccompanied, apart from two having their spouse with them. The interviews were semi-structured, following an interview guide (Supplement Table S1) including topics such as experiences and preferences of receiving information on disease and prognosis. The interviewers performed dialogue validation by paraphrasing and repeating the participant’s statements back to them to control for correct understanding [18]. Recruitment of participants continued until redundancy appeared, thereby reaching thematic saturation [11].

### Data analysis

The interviews were transcribed verbatim and analyzed by inductive qualitative content analyses without predefined categories [19, 20]. Through line-by-line analysis of the data, sections related to the research questions were identified and coded. The codes were sorted into categories and several categories were linked to different themes. Analysis of the transcripts, coding and development of categories was performed by two authors, AM and MK independently, cross-checked and when differing, discussed until agreement was reached.

## Results

### Participants

For participant demographics including MOCA, see Table 1. All were candidates for postoperative oncological treatment, either directly (for GBM) or at the time of tumor recurrence (for LGG). The MOCA score was within normal range for 15 participants. Ten had lower scores, indicating some cognitive impairment, and for these participants, questions could be adjusted. QoL data showed that participants were most troubled by fatigue and drowsiness and their role functioning was most affected (Supplement Figures S1a-c). There were no apparent differences between those with GBM or LGG, in neither MOCA nor QoL. We found, when analyzing the interviews and comparing all patients, that none of the identified categories were exclusive for one diagnosis or the other, younger versus older age, gender, education level or socio-economic status.

**Table 1:** Patient demographics

**Range**	
Age, years (median)	25–76 (51)
MOCA score (median)^a^	21–30 (26)
**Frequency**	
Diagnosis	
Glioblastoma (grade IV)	12
Anaplastic astrocytoma (grade III)^b^	1
Astrocytoma (grade II)^c^	3
Oligodendroglioma (grade II)^c^	9
Gender	
Male	15
Female	10
Marital status	
Married/cohabiting	18
Single/separated	6
Live-apart	1
Employment status	
Employed/self-employed	15
Unemployed	2
Medical discharge	1
Student	1
Retired	6
Survival status February 4th 2020	
Alive	13
Deceased	12

^a^MOCA (Montreal Cognitive Assessment) score: normal range 26–30

^b^One patient was diagnosed with anaplastic astrocytoma grade III, IDH wild-type and is referred to in the manuscript as high-grade glioma (HGG)

^c^Those diagnosed with either astrocytoma grade II or oligodendroglioma grade II are referred to as low-grade glioma (LGG) in the manuscript

### Analysis

In this paper, we present the analysis of the participants’ preferences and experiences of being informed about disease and prognosis (Table 2). They reported their experience of receiving information on the brain tumor, from when they contacted the health care with the first symptoms, to the definitive diagnosis and until the interview took place.

**Table 2:** Identified themes and categories

Diagnosis communication	Truth	Patients’ needs
• Indirect information • Insufficiently tailored information • Individualized and compassionate	• The good truth • The big picture • The whole truth	• Practical information • Supportive staff

## Themes

### Diagnosis communication

Information on diagnosis was most often received directly from a physician at a personal meeting, but not always. Most experienced getting a shock, a “knock-out”, when being told they had a brain tumor, while others already suspected the diagnosis. Some participants said that the behavior of the medical staff (e.g. facial expressions, body language or the instructions for the consultation) had prepared them for receiving news of having a serious illness. The experience of receiving information about the brain tumor could be divided into three categories:

#### Indirect information

Some participants found out they had a malignant brain tumour indirectly in unplanned ways, by reading the patient-accessed electronic health record (EHR) or when overhearing staff talking outside their room at the ward. In these situations, participants described feelings of fear, anxiety, abandonment and of being left alone with their questions, having no one to turn to. Some expressed regret about accessing the EHR.

(On accessing the EHR) *I just wanted to check out my medications. But then it just stood there! I would rather have liked to talk about it with the doctor first. What does this mean? You can´t take in that it says “malignant”! Who has written this? My whole body started to ache.* Woman, age 36, LGG

*I accessed the EHR and looked at the result - it’s called the preliminary result - and then I saw it. I felt… I felt it was a bit frightening // No, it wasn’t good. It’s destructive, self-destructive. You cannot handle it. You feel deserted with the information.* Man, age 70, GBM

#### Insufficiently tailored information

Many participants complained about lack of information due to delays, and the constant waiting and worrying were stressful. They also reported negative experiences when information was not adapted to their needs, preferences and timing, and when it contained too little or too much detail. Additionally, bad consultations were experienced when the physician appeared to be stressed or unprepared; was perceived as dishonest and hiding or omitting information; when the communication was unclear or when they did not seem competent regarding brain tumors. Some felt that their doctor was scared and avoided the topic, not being properly trained in delivering bad news.

*So I had to call and remind them. Call to ask, have you heard anything? You wait and wait and wait constantly, but you don’t want to nag, so you try to refrain [from calling], but eventually you can’t.* Man, age 52, LGG

(On experience of consultation) *Only quickly in, quickly out*. Woman, age 40, LGG

*It’s so difficult to stop and say: -Wait! I need further explanation on this, I need to understand.… The information was like this, pow, pow, pow, pow, pow.…it is not until you get home that you start thinking, how the hell is this going to work out?* (On providing negative information) *Doctors have to get rid of their fear for talking about things!* Man, age 67, LGG

#### Individualized and compassionate

Despite information being shattering, participants were content with direct information provided by the physician when it was given gradually, adapted to them and when the doctor was responsive and allowed for questions. It was also important that the doctor had knowledge in the field.

*Good straightforward information; they told me how it is, which was a bit tough, but they said: “This is it.” Did it in a good way, that I appreciated.* Man, age 56, GBM

*The doctor asked if I wanted a new appointment for follow-up questions, but as I already had brought a list of questions, he cancelled another meeting to be able to stay and answer them.... for me it was good.* Woman, age 36, LGG

*After the biopsy I e-mailed my neurosurgeon. He immediately called and told me what it was. (You got the diagnosis on the phone?) Yes… for me that was good. I have confidence in him.* Man, age 26, LGG

### Truth

All respondents stated that being told the truth about diagnosis and prognosis was important, and they did not want to be told lies. However, they had different perceptions on what constituted “the truth” and how it should be told. We identified three categories.

#### The good truth

A few participants stated that they wanted to know the truth, but only the good truth, meaning favourable information which would enable them to focus on what was positive. They felt it was better not to hear negative information, since receiving bad news could make them lose hope and become depressed. To cope with a poor prognosis, some wanted information to focus on short-term events, here and now. Even though they preferred not knowing the full prognosis, they still labelled information as “the truth” as opposed to being lied to.

*If something can be done, then I’d like to know, but otherwise I don’t want to know. I guess I’ll notice if it ends badly.* Woman, age 76, LGG

*Being told [about a poor prognosis] might just cause you to die faster, as you don´t see any hope. If you aren´t told, you can build up hope that makes you live longer. That is the reason I wouldn´t want to know.* Woman, age 45, GBM

#### The big picture

Some patients wanted truthful information on the overall prognosis, good or bad, but only the big picture. By not providing detailed information of the future and likely outcomes, hope could be preserved.

*Maybe you don’t have to go into the details, but say enough, so that you know the kind of general direction you´re heading.* Woman, age 36, LGG

*I want honest information, but if I want to know it all, that I don´t really know… that’s a difficult question to answer. Honest? It should be truthful but there must still be some hope.* Man, age 56, GBM

#### The whole truth

To have control, most respondents wanted complete and detailed information, even though it might hurt. Reasons given were to be able to accept their fate, plan ahead, inform family, decide about the near future, such as if they should accept a proposed treatment, or to be able to choose how to live the rest of their life. Some expressed that even though they wanted the full truth, they wanted to be told gradually, as they needed time to be prepared.

*I think the patient should have as much information as the doctor. There is no reason why the doctor should know more than me. Why should they have information that they haven’t shared with me?* Man, age 67, LGG

*I kind of wish I had a prognosis, but there is no one really who wants to say those things, I am not sure why. I guess they want to keep positive, but to me it is just always going to be ringing in the back of my head. I just want to know. You know, if I have, let´s say, hypothetically speaking, I have 10 years, then I don´t want to plan for 20, if I have 5 years, I don´t want to plan for 10 and so on…* Woman, age 26, HGG

*I think so [on wanting full information]. But I think I am a bit ambivalent also, because maybe not just now, but soon I think I´m getting ready for it. After all, you want to be able to plan your life. If you know that you only have a short time left, then I think it would become more important to be able to plan and focus the rest of your life on the right things.* Man, age 41, LGG

### Patients’ needs

Most participants described having little or no previous experience of illness and health care, and so after diagnosis, they reported being in an unfamiliar context, environment and role. What to expect was, for most, unknown, making them feel vulnerable. They expressed a need for more support and information than simply on diagnosis and prognosis.

#### Practical information

Participants experienced lack of practical information, like descriptions of procedures, risks, long-term plans, but also on smaller everyday things: When? Where? How? Why? What are the rules here, how am I supposed to behave? Participants described feeling they were disturbing staff and felt humiliated when having to ask questions repeatedly. Not knowing and not understanding caused an additional layer of distress, aggravated when health practitioners did not recognise this. Participants requested fast-track contact ways, assigned staff, simple but accurate and preferably written information.

*When I followed the doctor´s orders and called the ward about the problems with my medication, the nurse answered: “ Aha, what am I supposed to do about that?” I was so angry, so I just said: - You know what, I’ll fix this some other way. And then I hung up, and cried….* Woman, age 45, GBM

*I don’t understand how it’s organized. Therefore, I don’t know who to ask, and I suspect there is some kind of system. You lie there waiting, not knowing what you’re waiting for. It’s much better to be informed, to have practical information. To say, you have an X-ray today, but we don’t know when, it might be tonight, but we’re not sure. It means a lot. To say you don’t know is also information.* Man, age 56, GBM

#### Supportive staff

In order to handle the unknown, the support from staff was important. To be seen, listened to, feeling that someone cared. Knowing whom to contact when in need, being able to ask clarifying questions, was important. Some patients had been allocated a specialized brain tumor nurse, physician or social worker who stayed in touch and who they could turn to for support, making them feel safe and taken care of.

*I really don´t know what could have been better! The social worker has called me on the phone, nurses have called me, and all have been great!* Man, age 64, GBM

*In the radiation department a nurse came up to me and he recognized me! I really didn’t expect that when you haven’t had much contact. And it makes you really happy, it’s like being seen in a way.* Woman, age 61, GBM

*(Patient remembering a situation of despair) He spoke to me in a natural way// I felt I was part of life again. He didn’t talk about the illness, but instead about himself, his work and hobbies. Being able to talk to someone like that was wonderful//I almost cried.* Man, age 61, GBM

## Discussion

Our findings indicate that glioma patients have a need for individualized information regarding diagnosis and prognosis. In line with previous studies, those participating in our research expressed different preferences regarding how and when this should be provided as different patients need different levels of detail and timeframes to process mainly negative information [8, 21–23]. Also, our participants confirmed the importance of honest information, even though some wished not to be told if it was negative [21, 22, 24–26]. A study of terminally ill cancer patients also found different preferences for truthful communication of bad prognosis, varying from wanting the full truth, to a desired truth, this corresponding to different coping strategies [26]. Kirby found that patients’ response to a cancer diagnosis is not only shaped by the physician delivering the information, but also by patient factors when receiving information [27]. How information on diagnosis was received was influenced by the patient’s own social and life context: often shocking, sometimes surprising, in some confirming a suspicion and in others not only experienced as bad due to their life context. We also found all these response types in our study. This highlights the need for a responsive physician investigating patient context, in order to individually adapt communication.

We found that a mismatch between the patient’s preferences for information and what they experienced was provided by the physician, was not uncommon, which has been reported previously [28]. When informing about serious illness, the patient’s preferences are often not known, making the physician’s task difficult. A suggested solution is to simply ask the patient how much information he/she wishes to receive and provide honest and regarding content, individualized information [21, 22, 28]. Our participants appreciated being informed at their own pace and being able to have questions answered at follow-up consultations or phone calls with their physician or specialized nurse.

Some participants sensed fear and avoidance among physicians when delivering bad information. This experience is supported by previous reports [29–32]. These indicate that doctors hesitate to deliver bad news due to fears of eliciting emotional reactions in patients, fears of their own emotions being expressed and fears of being blamed personally for the bad news. Giving distressing information perceived as a “knock-out” triggers emotions not only in patients and family members, but also in physicians, who need support and training.

We found that health practitioners need to acknowledge patients’ requirements for everyday practical information, which, when lacking, adds to patients feeling lost and vulnerable. Recognition of the fact that patients are often novices in the health care system, and not familiar with matters self-evident for staff, needs to be improved. Apart from being allocated a specialized team member, who was easy to contact and get to know, some participants also felt they would get support from meeting other brain tumor patients.

In Sweden, the Brain Tumor Patient’s Association together with the health care has initiated several studies to improve patient care. These include communication between patients and the health care, patient and proxy needs and focus on the lived experience and psycho-social aspects. Additionally, Patient Reported Experience Measures (PREM) and Patient Reported Outcome Measures (PROM) are collected from all brain tumor patients for clinical routine and research [33]. These efforts hopefully contribute to better patient care and education of staff in the future.

Patient-accessed EHR has been launched to increase patient empowerment. Studies in patients with chronic illness show positive effects such as improved satisfaction, communication, engagement and self-care [34–37]. In Sweden, patient access to the EHR was introduced during the study period. Our study includes several examples of primary information on malignancy being indirectly, and unintentionally, communicated to patients when accessing their EHR, this seldom described in the literature. This highlights several negative consequences for patients, including increased anxiety, feeling deserted and regretting having accessed the EHR. It has been suggested that sensitive information could be flagged to make the patient aware of it, before accessing the EHR [36]. Alternatively, access to information could be delayed until communicated face-to-face. This was only partially implemented in the EHR system used by our participants. When designing and implementing systems for direct access of EHR, the needs of all type of patients and situations should be taken into account, for example by creating safe-guards, in order to avoid unnecessary harm to patients with newly diagnosed serious illness. More research is needed on the possible negative effects of patient-accessed EHR and how to minimize them.

### Limitations

We included participants from a defined region of Sweden and our findings may not be transferable to other regions or countries. The physicians informing participants were not interviewed so we cannot know if they experienced fear and avoidance, as perceived by some patients. Care was taken to include patients with different age, diagnosis, personal and socio-economic background. Despite this, it cannot be guaranteed that all perspectives were covered. Our study focused on those with glioma; therefore, we cannot know if our findings are valid for patients with other malignancies.

In qualitative studies, the researcher can affect interviewing and analysis of data; therefore, two researchers took part in the interviewing and analysis, in order to increase reflexivity. These are physicians with a long clinical experience of oncology and palliative care of glioma patients.

## Conclusion

To achieve patient-centred consultations, information on disease and prognosis but also on practical issues, needs to be adapted to each patient regarding amount, detail and timing, since patients have different individual preferences. This can be challenging for medical staff, whose need for training and support must not be overlooked. We suggest asking the patient directly about their preferences for information, since not everyone wants to know it all. Finally, how sensitive data are made available to patients needs to be thoroughly considered when developing and implementing patient-accessed EHR systems.

## Supplementary Information

ESM 1 (DOCX 13 kb)

ESM 2 (DOCX 160 kb)

## Data Availability

Not applicable.
