# Supplementary material for: “Do I want to know it all?” A qualitative study of glioma patients’ perspectives on receiving information about their diagnosis and prognosis
Source: Support Care Cancer. 2020 Oct 30;29(6):3339–46. doi: 10.1007/s00520-020-05846-7 (PMC8062391; doi:10.1007/s00520-020-05846-7)
Supplement: Supplementary file 1 — (DOCX 13 kb) [file 520_2020_5846_MOESM1_ESM.docx]

**Supplementary material**

“Do I want to know it all?”

A qualitative study of glioma patients’ perspectives on receiving information about their diagnosis and prognosis

Supportive Care in Cancer

Annika Malmström, Lisa Åkesson, Peter Milos, Munila Mudaisi, Helena Bruhn, Michael Strandeus, Marit Karlsson

**Corresponding author:** Annika Malmström, Department of Advanced Home Care, Linköping University, Sweden

Email: [Annika.malmstrom@regionostergotland.se](about:blank)

**Supplementary Table 1. Interview guide**

Background:

Age, profession

Disease:

Can you tell me about your disease? What happened? How did it all start? What happened after that? What have you found out?

Experience of health care:

What is your experience of the health care during this period?

Information about disease and treatment:

How did you get information about your disease? Was it clear? Good? Other wishes?

How did you get information about your treatment? Was it clear? Good? Other wishes?

Any suggestions for changes/improvement?

How were your proxies informed?

How would you have liked information to have been given- when? Where? By whom? With which participants?

Which type of information would you wish to receive?

Participation in decision making:

Do you feel you were part in the decision-making regarding your disease? How? Could you give an example? Was this good/bad? Other wishes?

How would you wish that decisions regarding your treatment were made? How much do you want to participate?

Prognosis:

If in the future it would be possible to find out the course of disease for each individual patient, how would you wish that this information was handled? How much would you like to know? If it was bad news? (How much do you think that your proxies would like to know?)

Do you have any additional questions or information you would like to share with me?
