# Supplementary material for: “Do I want to know it all?” A qualitative study of glioma patients’ perspectives on receiving information about their diagnosis and prognosis
Source: Support Care Cancer. 2020 Oct 30;29(6):3339–46. doi: 10.1007/s00520-020-05846-7 (PMC8062391; doi:10.1007/s00520-020-05846-7)
Supplement: Supplementary file 2 — (DOCX 160 kb) [file 520_2020_5846_MOESM2_ESM.docx]

**Supplementary material**

“Do I want to know it all?”

A qualitative study of glioma patients’ perspectives on receiving information about their diagnosis and prognosis

Supportive Care in Cancer

Annika Malmström, Lisa Åkesson, Peter Milos, Munila Mudaisi, Helena Bruhn, Michael Strandeus, Marit Karlsson

Corresponding author: Annika Malmström, Department of Advanced Home Care, Linköping University, Sweden

Email: [Annika.malmstrom@regionostergotland.se](about:blank)

**EORTC Quality of Life Questionnaire (QLQ30) and the Brain Cancer Module (BN20)**

We screened the participants in our study for factors that could influence the interview, such as symptom burden or cognitive decline. Therefore the EORTC QLQ30 and BN20 together with the MOCA test were conducted. For data on quality of life, we found that the participants in the study reported to be most troubled by fatigue (QLQ30) and drowsiness (BN20) and their most affected function was role functioning (QLQ30) (Supplementary Figures S1a-c below showing mean of all patients scale values (N=25)). There were no significant differences between those diagnosed with high grade glioma (HGG) (GBM and anaplastic astrocytoma, IDH wild type) or LGG. There was no correlation between cognition from MOCA and the patient´s own report of cognitive difficulties from the QLQ30 (p=0.74).


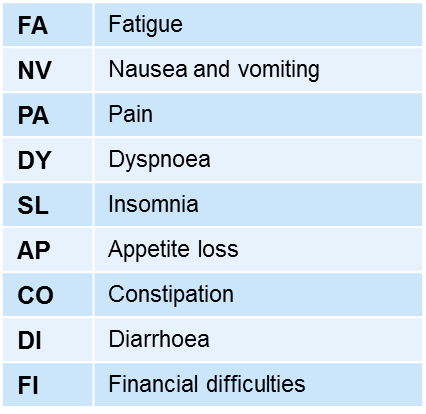


Figure S1a. Results for the symptom scales of the EORTC QLQ30 questionnaire, showing that fatigue was the most prominent symptom reported.


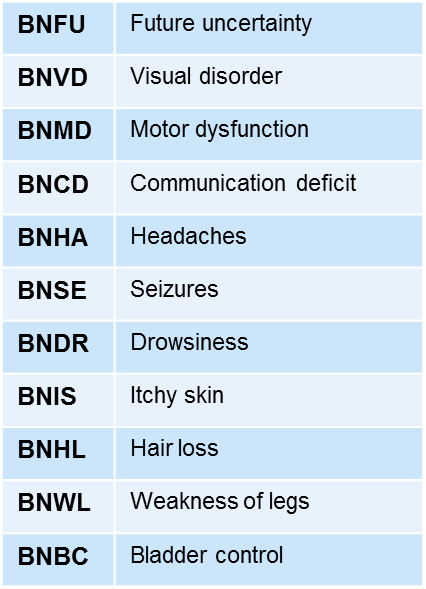


Figure S1b. Results for the symptom scales of the EORTC QLQ-BN20 questionnaire, showing that drowsiness was the most prominent symptom reported.


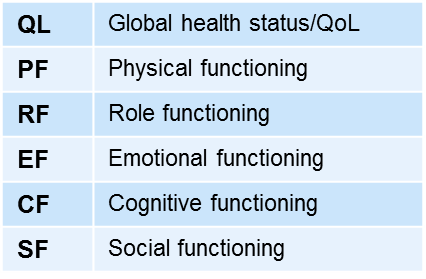


Figure S1c. Results for the functional scales of the EORTC QLQ-30 questionnaire, showing that Role functioning was reported as the most affected function (lowest value).
